# Urinary Bladder Dysfunction in Transgenic Sickle Cell Disease Mice

**DOI:** 10.1371/journal.pone.0133996

**Published:** 2015-08-04

**Authors:** Mário Angelo Claudino, Luiz Osório Silveira Leiria, Fábio Henrique da Silva, Eduardo Costa Alexandre, Andre Renno, Fabiola Zakia Mónica, Gilberto de Nucci, Kleber Yotsumoto Fertrin, Edson Antunes, Fernando Ferreira Costa, Carla Fernanda Franco-Penteado

**Affiliations:** 1 Department of Pharmacology, State University of Campinas, Campinas, SP, Brazil; 2 Hematology and Hemotherapy Center, State University of Campinas, Campinas, SP, Brazil; 3 Department of Clinical Pathology, Faculty of Medical Sciences, State University of Campinas, Campinas, SP, Brazil; 4 Laboratory of Multidisciplinary Research, São Francisco University Medical School, Bragança Paulista, SP, Brazil; Cinvestav-IPN, MEXICO

## Abstract

**Background:**

Urological complications associated with sickle cell disease (SCD), include nocturia, enuresis, urinary infections and urinary incontinence. However, scientific evidence to ascertain the underlying cause of the lower urinary tract symptoms in SCD is lacking.

**Objective:**

Thus, the aim of this study was to evaluate urinary function, *in vivo* and *ex vivo*, in the Berkeley SCD murine model (SS).

**Methods:**

Urine output was measured in metabolic cage for both wild type and SS mice (25-30 g). Bladder strips and urethra rings were dissected free and mounted in organ baths. In isolated detrusor smooth muscle (DSM), relaxant response to mirabegron and isoproterenol (1nM-10μM) and contractile response to (carbachol (CCh; 1 nM-100μM), KCl (1 mM-300mM), CaCl_2_ (1μM-100mM), α,β-methylene ATP (1, 3 and 10 μM) and electrical field stimulation (EFS; 1-32 Hz) were measured. Phenylephrine (Phe; 10nM-100μM) was used to evaluate the contraction mechanism in the urethra rings. Cystometry and histomorphometry were also performed in the urinary bladder.

**Results:**

SS mice present a reduced urine output and incapacity to produce typical bladder contractions and bladder emptying (*ex vivo*), compared to control animals. In DSM, relaxation in response to a selective β3-adrenergic agonist (mirabegron) and to a non-selective β-adrenergic (isoproterenol) agonist were lower in SS mice. Additionally, carbachol, α, β-methylene ATP, KCl, extracellular Ca^2+^ and electrical-field stimulation promoted smaller bladder contractions in SS group. Urethra contraction induced by phenylephrine was markedly reduced in SS mice. Histological analyses of SS mice bladder revealed severe structural abnormalities, such as reductions in detrusor thickness and bladder volume, and cell infiltration.

**Conclusions:**

Taken together, our data demonstrate, for the first time, that SS mice display features of urinary bladder dysfunction, leading to impairment in urinary continence, which may have an important role in the pathogenesis of the enuresis and infections observed the SCD patients.

## Introduction

Sickle cell disease (SCD), an inherited disorder of hemoglobin synthesis, is caused by a single nucleotide substitution (GTG for GAG) in the sixth codon of the β-globin gene. This mutation results in the substitution of valine for glutamic acid on the surface of the variant β-globin chain [1]. The multiple pleiotropic effects of the abnormal hemoglobin S production include vaso-occlusive crisis, stroke, pulmonary hypertension, osteonecrosis, leg ulcers and priapism [1–3]. SCD-associated urological complications have also been described, such as nocturia, enuresis, increased frequency of urinary infections and urinary incontinence [4]. Among the SCD patients presenting urinary tract infection, one to two thirds exhibit recurrent infection that may be accompanied by fever [5]. There is also an increased incidence of urinary tract infection during pregnancy in sickle cell trait and SCD [6–9]. As part of the renal complications of sickling, renal medullary infarcts lead to decreased ability to concentrate urine, yielding higher daily urinary volumes [10], compensatory polydipsia, and possibly the need for nocturnal bladder voiding [4]. Additionally, in SCD, there is a strong association between enuresis and overactive bladder symptoms such as daytime incontinence, urgency and frequency [11–15]. However, scientific evidence to ascertain the underlying cause of the lower urinary tract symptoms (LUTS) in SCD patients is lacking. Some hypotheses to explain LUTS in these patients include inability of the kidneys to concentrate urine, social and genetic factors, delays in neurophysiological development, and urinary bladder dysfunction [16, 17] [4, 18].

The abilities of the lower urinary tract to store and to release urine are regulated by neural circuits located in the brain, spinal cord and peripheral ganglia. The sacral parasympathetic outflow provides the main excitatory input to the urinary bladder via the release of both cholinergic and non-adrenergic, non-cholinergic transmitters [19–21]. Detrusor smooth muscle (DSM) expresses muscarinic M2 and M3 receptors in a variety of animal species, but M3 receptors have been reported to be functionally more important for urinary bladder contractions and efficient emptying than M2 receptors [22, 23]. Non-cholinergic excitatory transmission mediated by ATP via purinergic P2X receptors in DSM may also contribute to bladder contractions [24]. Sympathetic innervation of the bladder arises in the thoracolumbar outflow of the spinal cord and releases noradrenaline, which activates inhibitory β-2 and β-3 adrenoceptors in DSM, causing bladder relaxation and contributing to the urine storage phase [19]. In addition, sympathetic stimulation will also stimulate α1-adrenoceptors in the urethra to provide bladder outlet resistance and prevent involuntary leakage of urine [25]. Changes to the contractile and relaxant mechanisms of the urethra and DSM may lead to either overactive or underactive bladder, depending on the stage of the disease [26].

Several murine models have been developed to mimic human SCD. The Berkeley murine model of SCD which expresses human sickle hemoglobin exclusively is a well-accepted animal model that displays most of the clinical features of humans SCD [27, 28], including priapism, the most prominent urogenital manifestation of SCD. Since little is known of the pathophysiology of other urogenital disorders in SCD, we have sought to use the same animal model to gather further information on how SCD affects bladder function. Therefore, in the present study, we have used the Berkeley murine model to characterize DSM dysfunction *in vivo*, investigate urethra contractile response, receptor-dependent and receptor-independent contractile- and relaxant-responses in the DSM *ex vivo*, and urinary bladder structure in SCD.

## Materials and Methods

### Ethics Statement

All experimental procedures in this study were carried out in accordance with the general ethical guidelines for animal use established by the Brazilian Society of Laboratory Animal Science (SBCAL) and EC Directive 86/609/EEC for Animal Experiments and were approved by an institutional Committee for Ethics in Animal Experimentation of the University Of Campinas (IACUC/CEEA-UNICAMP, Permit number 1864–1). The animals were euthanized with an overdose of sodium pentobarbital (1mg/g) and all efforts were made to minimize animal suffering.

### Animals

Berkeley transgenic sickle cell mice (SS mice), expressing exclusively human sickle hemoglobin ((Tg(Hu-miniLCRα1GγAγδβS)Hba0//Hba0Hbb0//Hbb0]; [27]) and C57BL/6 control mice were used for these studies (20–25 g). The animals were obtained from Jackson Laboratories (Bar Harbor, ME, USA) and were generated and characterized at the Multidisciplinary Center for the Investigation of Biological Science in Laboratory Animals of State the University of Campinas (UNICAMP) and were maintained at constant room temperature on a 12 h light/dark cycle, with free access to food and water.

### Urine output measurement in metabolic cage

Mice (4 in each group) were individually housed in metabolic cages (Cat No. 3701M081, Tecniplast, Buguggiate, VA, Italy) with a floor area of 200 cm^2^ (dimensions 32 X 25 X 36.5 cm, length X width X height). Mice were fed and received water *ad libitum* and, after 24 h in the metabolic cages, weight and 24h urinary volume were recorded.

### *In vivo* cystometry

Mice were anaesthetized with intraperitoneal urethane (1.8 g/kg). Once surgical anesthesia was reached, a 1 cm incision was made along the midline of the abdomen. The bladder was exposed and a butterfly cannula (25 G) was inserted into the bladder dome. The cannula was connected to a three-way tap. One port was connected to a pressure transducer and another to an infusion pump through a catheter (PE50). Before starting cystometry, the bladder was emptied through the third port. Continuous cystometry was performed by infusing saline into the bladder at a rate of 0.6 mL/h. The following parameters were assessed: threshold pressure (TP; intravesical pressure immediately before micturition); post-void pressure (PVP; intravesical pressure immediately after micturition); peak pressure (PP; highest intravesical pressure during micturition); capacity (CP; volume of saline needed to induce the first micturition); compliance (CO; CP to TP ratio); frequency of voiding contractions (VC) and frequency of non-voiding contractions (NVCs). NVCs were defined as spontaneous bladder contractions greater than 4 mmHg from the baseline pressure that did not result in micturition. Bladders from mice used for cystometry were not used in other experiments.

### *Ex vivo* functional studies

The urinary bladder and urethra were surgically removed and placed in chilled Krebs-Henseleit buffer (118mM NaCl, 25mM NaHCO_3_, 5.6mM glucose, 4.7mM KCl, 1.2mM KH_2_PO_4_, 1.17mM MgSO_4_.7H_2_O and 1.17mM CaCl_2_.2H_2_O). After removal of the trigone, the DSM was cleaned of connective and adventitial tissues, and two strips (1.0 x 0.2 x 0.2 cm) of bladder were obtained from each animal. The strips had intact urothelium, and the urethra was removed and cut into rings (1 to 1.5 mm in length). The DSM strips were mounted in a 10mL organ system and the urethra rings were mounted in 5mL organ baths, both containing Krebs solution at 37°C, that was continuously bubbled with a mixture of 95% O_2_ and 5% CO_2_. DSM strips were vertically suspended between two metal hooks. One hook was connected to a force transducer (Ugo Basile, Varese, Italy) and the other acted as a fixed attachment point. Tissues were allowed to equilibrate for 60 min under a resting tension of 5 mN. For the urethra rings, the resting tension was adjusted to 2 mN at the beginning of the experiments. The equilibration period was 45 minutes, and the bathing medium was changed every 15 minutes until the start of the experiment. Changes in isometric force were recorded using a Power Lab v.7.2 system (ADInstruments, Sydney, Australia).

### Concentration–response curves

To verify the viability of the preparations, high extracellular K^+^ solution (80 mM, achieved by substituting NaCl in Krebs buffer for an equimolar concentration of KCl) was added to the baths at the end of the equilibration time. To evaluate the contraction mechanism of DSM strips, the full muscarinic agonist carbachol (0.01 to 100 μM) and a hyperpolarizing solution of KCl (1 to 300 mM) were used to obtain cumulative concentration-response curves in half-log steps. Non-cumulative concentration–response curves to the purinergic P2X receptor agonist, αβ-methylene ATP (1, 3 and 10 μM), were also obtained. For the evaluation of the relaxation mechanism, cumulative concentration–response curves to the non-selective β-adrenergic agonist isoproterenol (0.001–10 μM), and the selective β3-adrenoceptor agonist, mirabegron (0.001–10 μM), were obtained from DSM strips precontracted with carbachol (1 μM for control mice and 3 μM for SS mice). To evaluate the contraction mechanism of urethra rings, the α1-adrenoceptor agonist phenylephrine (0.01 to 100 μM) was used to obtain cumulative concentration-response curves in half-log steps. Nonlinear regression analysis to determine the potency (pEC_50_) was performed using GraphPad Prism (GraphPad Software, San Diego, CA, USA) with the constraint that Φ = 0. All concentration–response data were evaluated for a fit to a logistics function in the following formula:

$$E=E_{\text{max}}/[1+{\left(10^{\text{c}}/10^{\text{x}}\right)}^{\text{n}}]+\Phi$$

where *E* is the maximum response produced by agonists; *c* is the logarithm of the EC_50_, the concentration of drug that produces a half-maximal response; *x* is the logarithm of the concentration of the drug; *n* is a curve-fitting parameter that defines the slope of the concentration response line; and Φ is the response observed in the absence of added drug. Values of pEC_50_ and maximal response (E_max_) were expressed as mean ± SEM of N experiments. E_max_ data were normalized to the wet weight of the respective urinary bladder strips, and the values of E_max_ were represented as mN per milligram wet weight.

### Concentration response curves to extracellular CaCl_2_

To evaluate the receptor-independent, direct effects of extracellular Ca^2+^ influx on bladder contraction, cumulative concentration response curves to CaCl_2_ (0.01 to 100 mM) under depolarizing conditions were obtained. Bladder strips were prepared and mounted in 10mL organ baths containing Krebs–Henseleit Ca^2+^-free solution containing 1mM EGTA to sequester Ca^2+^ ions, and cyclopiazonic acid (CPA, 10 μM) to deplete sarcoplasmic reticulum Ca^2+^ stores. Bath solution was removed and replaced by Krebs–Henseleit Ca^2+^-free solution containing KCl (80 mM) and CPA (10 μM). After an equilibration period of 15 min, the cumulative curve to CaCl_2_ was obtained [29].

### Electrical field stimulation

Electrical field stimulation (EFS) was applied to bladder strips placed between two platinum ring electrodes connected to a Grass S88 stimulator (Astro-Med Industrial Park, West Warwick, RI, USA). EFS was conducted at 80 V, 1 ms pulse width and trains of stimuli lasting 10 s at varying frequencies (1–32 Hz), with 3 min interval between stimulations. Subsequently, after incubation periods of 30 min, frequency–response curves were repeated in the presence of the muscarinic receptor antagonist atropine (1 μM) and/or the P2X receptor blocker suramin (100 μM), to confirm that the responses were mediated by muscarinic (acetylcholine; ACh) and P2X (ATP) receptor activation (B; [29]).

### Histological Analysis

The animals were euthanized by deep anesthesia before removal of the bladder. Bladders were split longitudinally for histological processing. The tissues were fixed in 10% formaldehyde solution for 24 h and subsequently dehydrated in a graded ethanol and xylol series, then embedded in Histosec (Merck, Rio de Janeiro, RJ, Brazil). Sample tissue sections of 5 μm thick were deparaffinized and stained with hematoxylin and eosin for examination by light microscopy. Histological analyses were performed by two pathologists blinded to the origin of the specimens.

### Drugs

Atropine, carbachol, cyclopiazonic acid, isoflurane, isoproterenol, mirabegron, phenylephrine, suramin, urethane and α,β-methylene ATP were obtained from Sigma Chem Co. (St. Louis, MO, USA).

### Statistical analysis

Data are expressed as mean ± SEM of n experiments. In the non-cumulative-, cumulative-concentration- and frequency–response curves, data are expressed as means of the contraction in mN·mg^-1^ of wet strip weight ± SEM of n experiments to control for differences in size between bladder strips. Statistical software Instat (GraphPad Inc., USA) was used for statistical analysis. Two-way analysis of variance and Student’s t-test were used to evaluate the results. *P* < 0.05 was accepted as significant.

## Results

### Urine output is reduced in SS mice

After 24 h of restraint in the metabolic cages, water intake did not change in SS, compared with the control group (n = 4, respectively; data not shown). However, SS mice presented a significant reduction in urine output (0.92 ± 0.23 ml), when compared to control mice (1.98 ± 0.38 ml).

### Cystometry reveals that SS mice exhibit an underactive bladder

**Fig 1:**
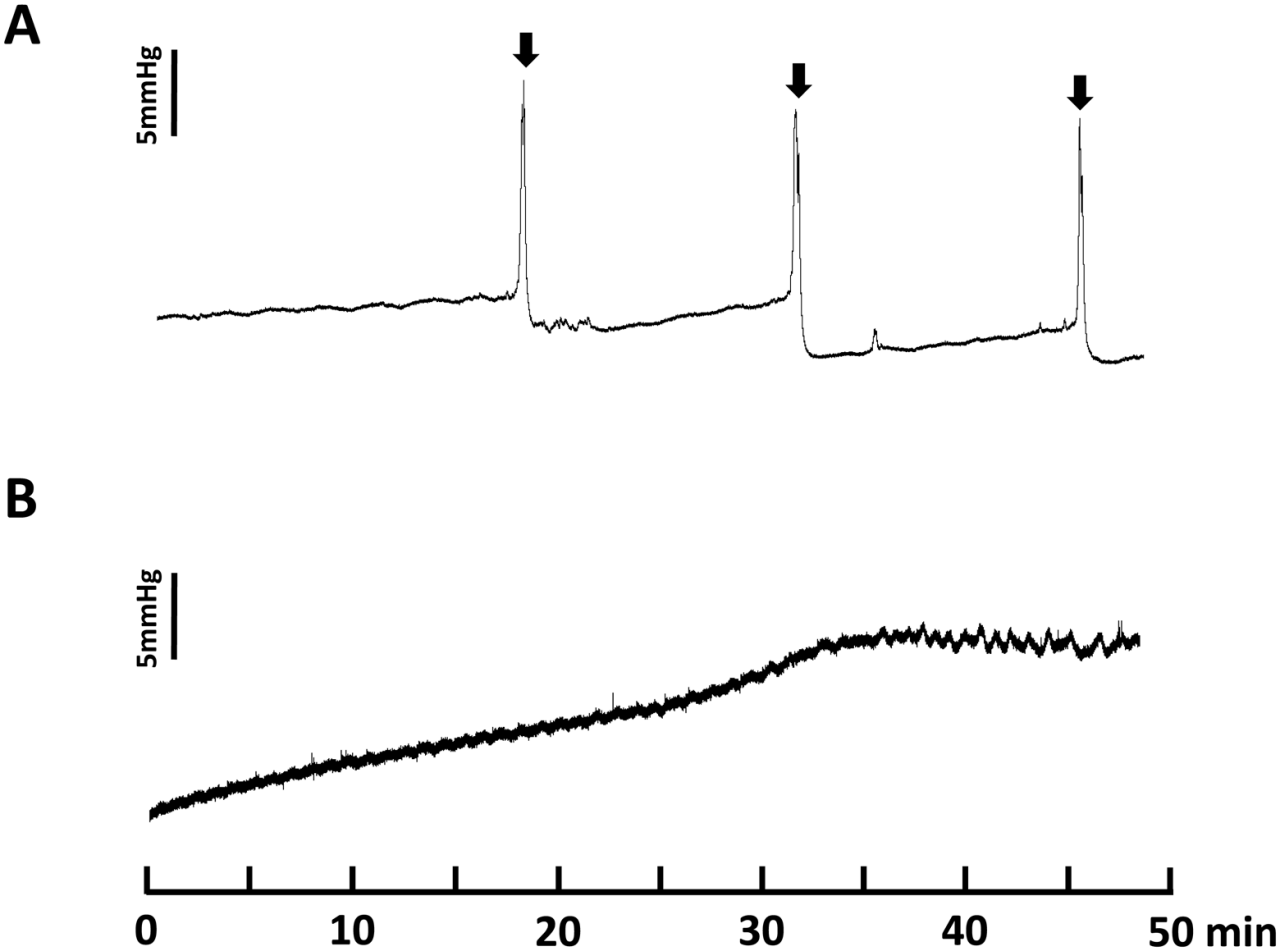
Representative cystometric recording from control (A) and Berkeley transgenic sickle cell (SS) mice (B). Arrows in the cystometric trace indicate the micturition peaks.

In the cystometric study, control mice showed regular micturition cycles with rare non-voiding contractions (n = 8; Table 1; Fig 1A). All of the parameters measured were comparable with our previous data for cystometry in control mice (Leiria et al., 2011). However, homozygous SS mice showed an atypical voiding pattern characterized by an incapacity to produce typical bladder contractions and bladder emptying during a 50-min observation. As shown in Fig 1B, the intravesical pressure during the filling phase increased progressively until reaching a pressure that caused urine leakage (n = 7; Table 1).

**Table 1:** General, cystometric and morphologic parameters for control and SS mice.

Parameters	Control	Homozygous (SS)
Body weight (g)	24.6 ± 1.54	25.4 ± 1.67
**Cystometric data**		
Threshold Pressure (ThP, mmHg)	6.25 ± 0.82	-
Capacity (mL)	0.24 ± 0.03	-
Compliance (mL/mmHg)	0.027 ± 0.002	-
Peak Pressure (mmHg)	12.45 ± 1.69	-
Frequency (n° of voidings/min)	0.18 ± 0.03	-
NVCs (n° of NVCs/min)	0.07 ± 0.03	-
Post Void Pressure (mmHg)	0.90 ± 0.53	-
**Morphologic data**		
Urinary bladder weight (mg)	26.6 ± 1.55	17.5 ± 2.21 **
Urothelium thickness (mm)	0.041 ± 0.004	0.031 ± 0.002
Detrusor thickness (mm)	0.37 ± 0.04	0.26 ± 0.03 **
Bladder volume (mm^3^)	17.8 ± 1.83	4.8 ± 0.55 **

Data represent the means ± SEM for 8–14 mice.

** P<0.01.

### Bladders from SS mice have reduced relaxation and contraction response *ex vivo*

**Fig 2:**
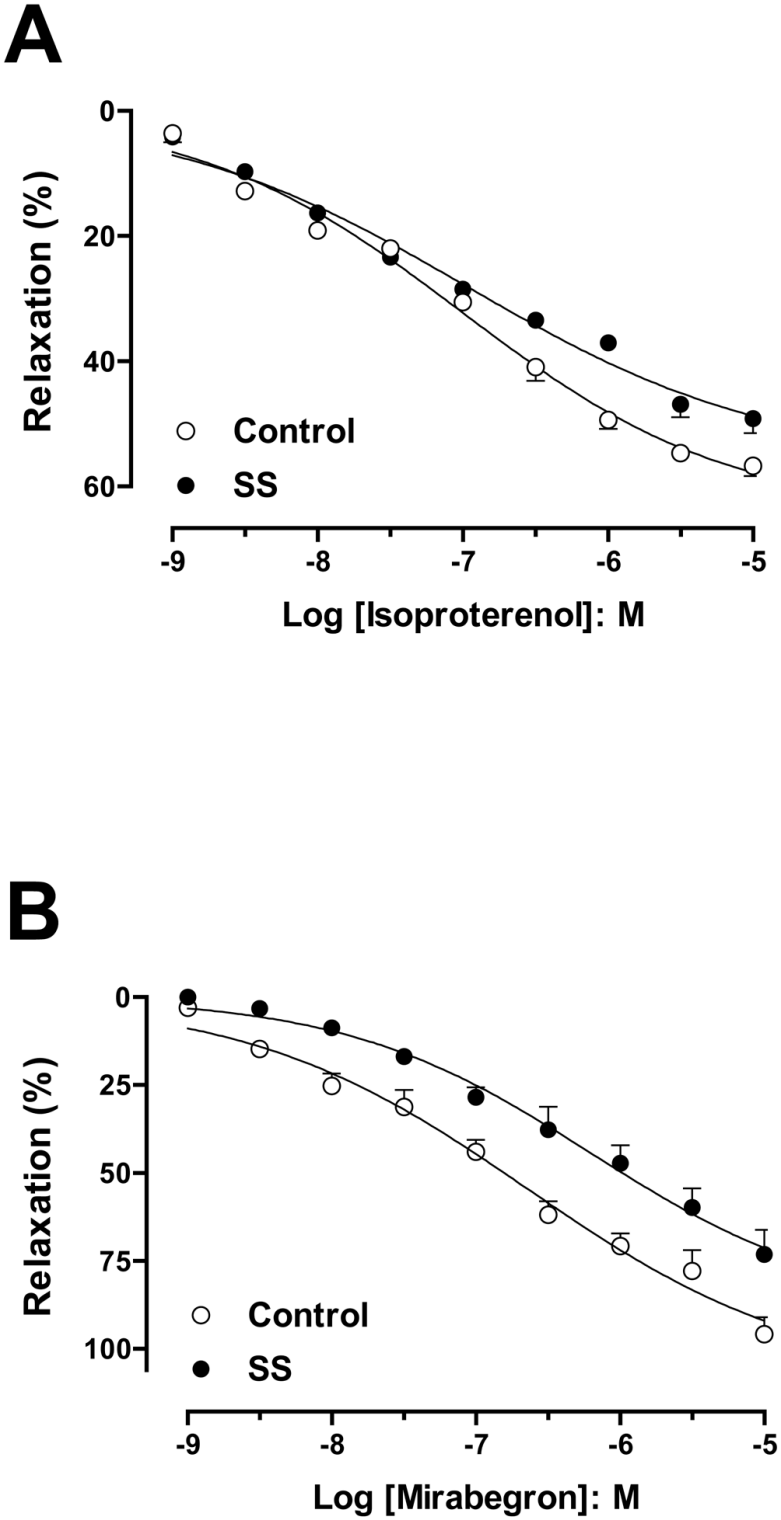
Concentration-response curves from isoproterenol (A) and mirabegron (B) in detrusor smooth muscle strips from control (A) and Berkeley transgenic sickle cell (SS) mice (B) precontracted by carbachol (1 μM for control mice and 3 μM for SS mice). Experimental values were calculated relative to the maximal changes from the contraction produced by carbachol in each tissue, which was taken as 100%. Data represent the means ± SEM of N experiments. *P < 0.05 compared with control group.

The cumulative addition of the isoproterenol or mirabegron produced concentration-dependent DSM relaxations in both control and SS groups (Fig 2A and 2B). Relaxation responses both to isoproterenol and mirabegron were significantly lower in DSM from SS mice (Emax: 49.1 ± 2.3%; Emax: 73.2 ± 7.0%; n = 4; P<0.05, respectively), when compared with the respective control mice (Emax: 56.7 ± 1.6%; Emax: 95.8 ± 4.8%; n = 4). No differences in the pEC50 values for isoproterenol or mirabegron were found between the control (7.00 ± 0.12; 6.64 ± 0.20; n = 4, respectively) and SS groups (6.94 ± 0.19; 6.20 ± 0.24; n = 4, respectively) (Fig 2A and 2B).

**Fig 3:**
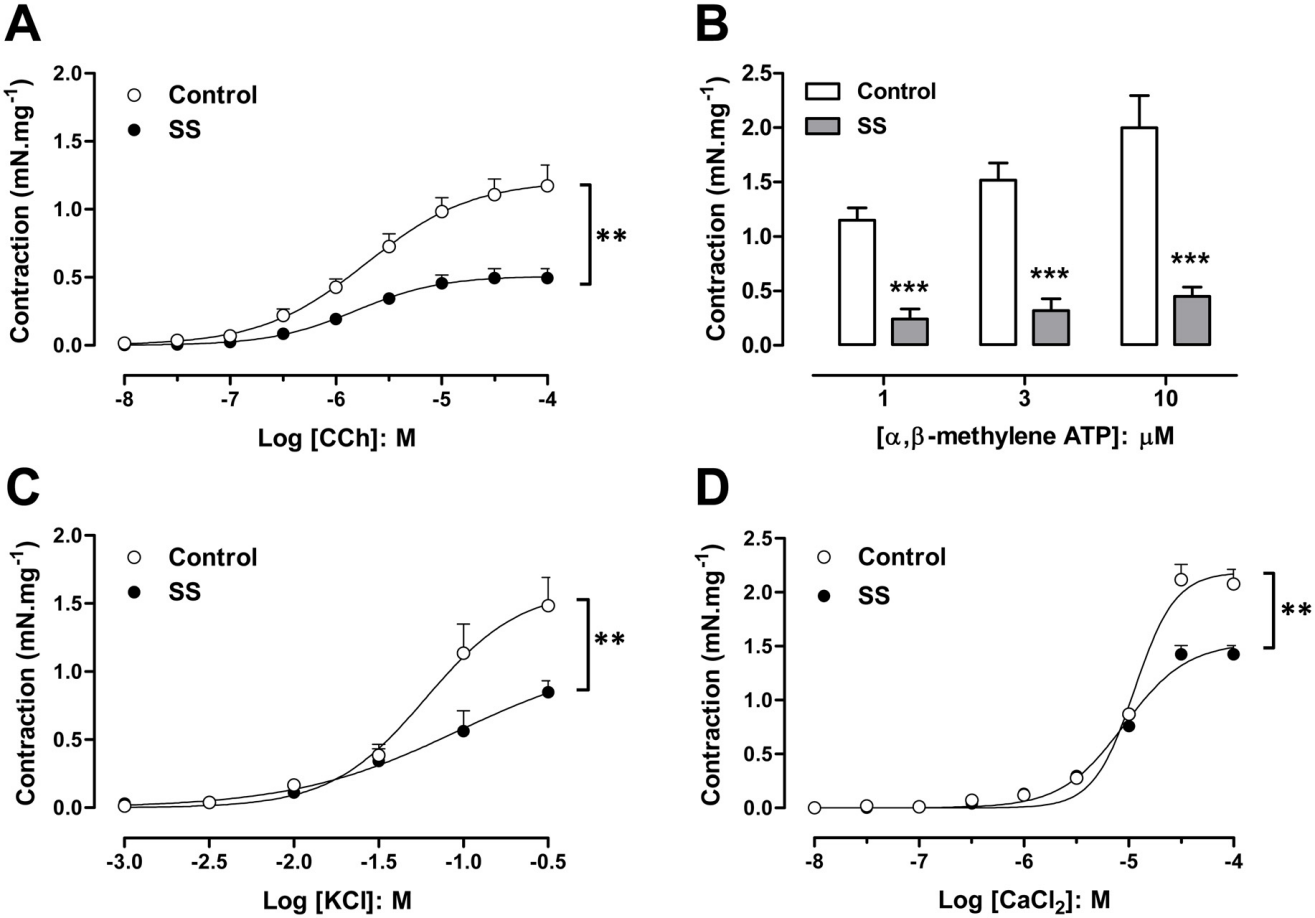
Detrusor smooth muscle contraction in response to the muscarinic agonist carbachol (A), the P2X agonist α,β-methylene ATP (B), potassium chloride (C) and calcium chloride (D) in bladder strips from control and Berkeley transgenic sickle cell (SS) mice. Data represent the means ± SEM of N experiments. **P < 0.01; ***P < 0.001 compared with control group.

Both the muscarinic agonist (carbachol) and the P2X receptor agonist (α, β methylene ATP) produced concentration-dependent DSM contractions in the control and SS groups (Fig 3A and 3B). However, maximal carbachol-induced DSM contractions were markedly lower in SS mice (0.49 ± 0.06 mN.mg^-1^; n = 9; P<0.01), when compared with the control group (1.18 ± 0.15 mN.mg^-1^; n = 6). No differences in the potency (pEC_50_) values for carbachol were found between the control (5.70 ± 0.17; n = 6) and SS (5.81 ± 0.23; n = 9) groups. A similar reduction was seen with the maximal response to α, β methylene ATP (P<0.001, n = 4 for each group).

In the evaluation of receptor-independent stimulation, cumulative addition of KCl produced concentration-dependent DSM contractions. The maximal contractions were significantly lower in DSM from SS mice (Emax: 0.85 ± 0.08 mN.mg^-1^; n = 8; P<0.01), when compared with the control mice (Emax: 1.48 ± 0.20 mN.mg^-1^; n = 8; Fig 2C). No differences in the pEC_50_ values for KCl were found between the control (1.22 ± 0.13; n = 8) and SS groups (1.04 ± 0.20; n = 8; Fig 3C). DSM maximal contractions to CaCl_2_ were significantly reduced in strips from SS mice (1.42 ± 0.08 mN.mg^-1^; n = 4; P<0.01), compared with those from control mice (2.07 ± 0.13 mN.mg^-1^; n = 4; Fig 3D). No differences in the pEC_50_ values for CaCl_2_ were found between the control (4.94 ± 0.03; n = 4) and SS groups (5.04 ± 0.05; n = 4; Fig 3D).

### Urethra from SS mice have a reduced contractile response *ex vivo*

The cumulative addition of the phenylephrine produced concentration-dependent urethra ring contractions in both the control and SS groups. However, maximal phenylephrine-induced urethra contractions were markedly lower in SS mice (25.01 ± 8.68 mN.mg^-1^; n = 4; P<0.001), when compared with the control group (89.47 ± 2.35 mN.mg^-1^; n = 6; data not shown).

### The DSM response to electrical-field stimulation is reduced in SS mice and is partially mediated by acetylcholine and ATP

**Fig 4:**
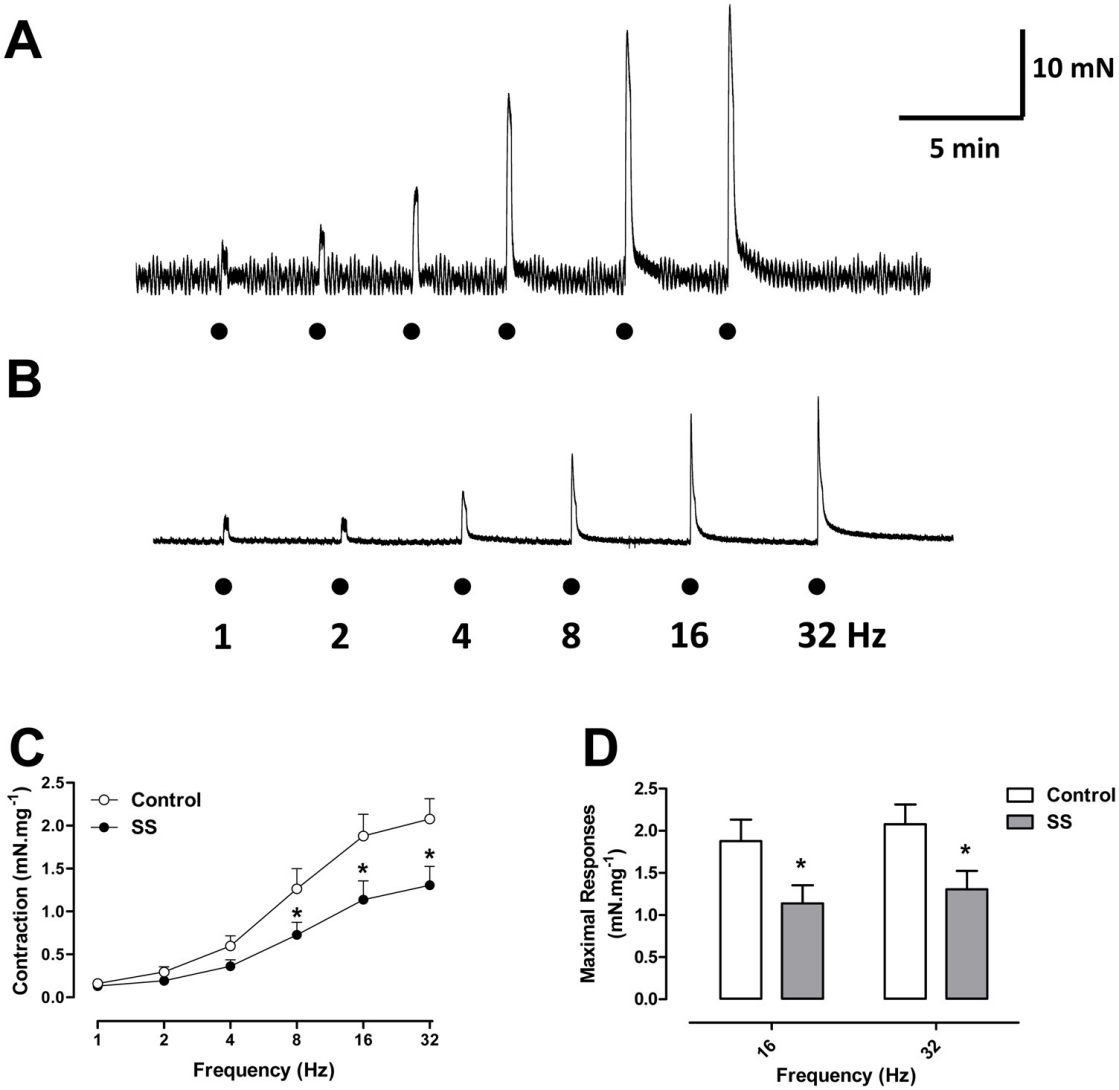
Representative trace of a frequency-response curve to EFS in detrusor smooth muscle from (A) control and (B) Berkeley transgenic sickle cell (SS) mice in basal tonus. Frequency-response curves to electrical field stimulation (EFS; 1–32 Hz) (C) in detrusor smooth muscle strips from control (open circles) and Berkeley transgenic sickle cell (SS; closed circles) mice in basal tonus. Maximal response values are represented in panel D. Data represent the means ± SEM of N experiments. *P < 0.05 compared with control group.

Electrical-field stimulation (EFS) produced frequency-dependent DSM contractions in both groups, which were significantly reduced in the SS group at the highest frequencies employed (16 and 32 Hz; P<0.05; Fig 4). Pretreatment of DSM preparations with the muscarinic and purinergic receptor combined reduced the EFS-induced DSM maximal contractions by approximately 60% in both groups studied (P<0.01). In controls, maximal contractions decreased from 23.8 ± 3.8 to 10.36 ± 1.9 mN (n = 5), and in SS mice, from 11.8 ± 2.1 to 5.2 ± 1.4 mN (n = 6), confirming that EFS-elicited contractions are mediated, in part, by ACh and ATP.

### Urinary bladders of SS mice suffer morphological changes

**Fig 5:**
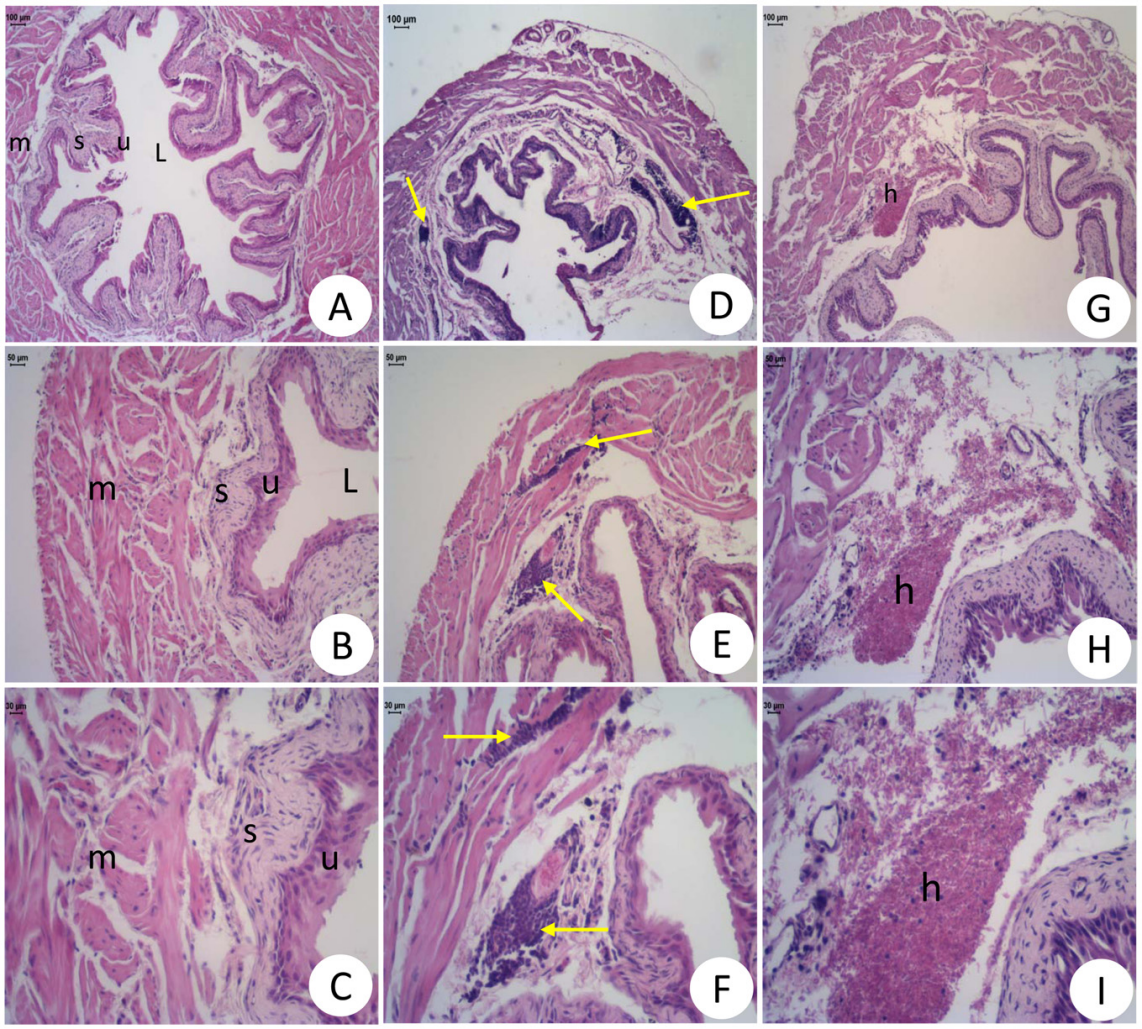
Histological sections of urinary bladder wall in normal (A-C) and Berkeley transgenic sickle cell (SS) mice (D-I). U = urothelial cells; S = submucosa; L = lumen; M = smooth muscle; E = edema; H = hemorrhage; Yellow Arrows: inflammatory cells. Bar = 50 μm (B, E and H) and 30 μm (C, F and I). Hematoxylin and eosin staining.

Light microscopy of bladders from normal mice confirmed an absence of structural abnormalities (Fig 5A and 5B). Bladders from SCD mice presented with severe lesions and significant reductions in detrusor thickness and bladder volume, when compared to those of control mice (Table 1). Infiltration by inflammatory cells (mononuclear cells) in the submucosa and muscular area was also seen in the tissue of SCD mice (Fig 5C and 5D). In some samples, there was also the presence of hemorrhage (Fig 5E and 5F) and edema (Fig 5C–5F).

## Discussion

Urogenital disorders in SCD have been extensively explored both in the clinic and in experimental research regarding the pathophysiology and treatment of priapism, both in humans and in mouse models. Nonetheless, urinary bladder dysfunction (UBD) has not been the focus of extensive research, and clinical data on this matter is scant. Our data show for the first time that, in addition to chronic hemolytic anemia, vaso-occlusion, and priapism, the Berkeley SCD mouse model also exhibits a characteristic UBD.

The urinary bladder has two important functions: urine storage and emptying, and both of these seem to be abnormal in SS mice. A main physiological mechanism to induce DSM relaxation needed to allow bladder filling under low pressure is β-adrenoceptor stimulation, which, in most species including humans, involves a strong β3-component [25]. The reduced response to selective (mirabegron) and non-selective (isoproterenol) beta-adrenergic stimulation in bladder strips from SS mice suggests that relaxation is reduced in this model.

Micturition occurs when sympathetic fibers are activated during the filling process and the central nervous system recognizes bladder fullness switching from the stimulation of sympathetic fibers to parasympathetic fibers [30]. ATP is co-stored and released together with the main neurotransmitter acetylcholine (Ach) [31, 32], contributing to nerve-mediated bladder contraction and urine elimination [33, 34]. Acetylcholine receptor M3 and ionotropic P2X1 receptors are responsible for the contractile response in the urinary bladder [20]. Our data show that both muscarinic and purinergic blockade reduce, but do not abolish DSM contraction, highlighting DSM hypoactivity, but also corroborating the existence of a muscarinic- and purinergic-resistant neurogenic component in the bladder that has not yet been fully characterized [35, 36].

Both calcium and potassium are involved in receptor-independent muscle contraction. M3 receptors also interact with Gq to elicit the release of Ca^2+^ from internal stores through inositol-1,4,5-trisphosphate (IP_3_) receptor activation [20], and muscarinic agonist induced contractions also partly depend on Ca^2+^ entry through Cav1.2 channels [30, 37–40]. High levels of extracellular K^+^ depolarize the cell membrane and activate Cav1.2 channels, resulting in an increased inward movement of Ca^2+^, which in turn activates contractile proteins [19]. Our findings show that DSM from SS mice present a reduction in the contractile response to both KCl and extracellular CaCl_2;_ suggesting that reduced extracellular Ca^2+^ entry via Cav1.2 channels plays a critical role in the underactive DSM.

Reduced contractile responses to muscarinic and purinergic agonists, KCl, CaCl_2_, and EFS, with impairment of the relaxation mechanism induced by isoproterenol and mirabegron, corroborate the hypothesis that these animals have an atonic detrusor smooth muscle. This pharmacological characterization of major mechanisms involved in the DSM atonia of SS mice (*ex vivo*) provides pathophysiological support for the alterations in the contraction or relaxation mechanisms of DSM during the filling and emptying phases observed *in vivo*: these animals have a reduced urinary volume, suggesting urinary retention, and their urodynamic parameters show absence of urinary bladder activity. Histomorphometric analysis revealed significant changes in SS mice bladder structure, such as reduction of the bladder wall thickness and volume, along with reduction of wet weight. Decrease in wall thickness may explain the impairment in bladder contractile ability, since a reduction in muscle thickness probably affects muscle contraction. Thus, SS mice probably have an underactive urinary bladder due to atonic DSM.

While ischemia-reperfusion injury occurs in SCD, we are not aware that chronic ischemia of the urinary bladder has been described in these patients. Regarding mechanisms that could explain this bladder phenotype, there is evidence that chronic bladder ischemia can cause bladder dysfunction in atherosclerosis of the pelvic vessels [41]. This type of damage has been attributed to impaired perfusion, leading to oxidative stress of the bladder wall. Excessive reactive oxygen species production is a pathophysiological mechanism common to both atherosclerosis and SCD, and progression from moderate to severe bladder ischemia in atherosclerosis is thought to cause overactive bladder syndrome in elderly patients that later progresses to symptoms of underactivity [42]. Since SCD vaso-occlusion causes repeated cycles of ischemia-reperfusion injury from birth, bladder ischemia might ensue earlier and more severely than in acquired progressive ischemia, possibly explaining our finding of an underactive bladder in SS mice.

Despite SS mice exhibiting an underactive bladder and severe structural abnormalities, our analysis also revealed presence of hemorrhage, edema, and submucosal and muscular infiltration by mononuclear cells. The presence of inflammatory cells in the muscular and submucosa layers of the bladder wall in SS mice supports the existence of a chronic inflammatory state of the urinary bladder, which may be a morphological finding corresponding to the occurrence of oxidative stress in the bladder a pathological mechanism underlying bladder dysfunction in other models, such as chronic bladder ischemia in atherosclerosis [41].

UBD is rarely spontaneously reported by SCD patients to their caregivers, but clinical observations of medical complaints involving the urinary bladder start as early as childhood, with enuresis, and continue onto adulthood with nocturia and urinary tract infections (UTIs) [4, 14]. SCD-associated UBD may contribute to impair the bladder emptying function leading to the presence of residual urine. It is known that residual urine in a high-pressure container causes either decompensation of the detrusor with vesicoureteral reflux or deterioration of the bladder wall with hypertrophy and stiffness resulting in ureterovesical obstruction. The subsequent insufficient drainage of the upper urinary tract leads to decompensation of the ureters and finally to chronic renal disease, the process being accelerated by urinary tract infections (UTI; [43]). An epidemiological study of 321 children with SCD showed that 7% had a documented UTI, one third had recurrent infections, and two-thirds had a febrile UTI [5], and most episodes of gram-negative septicemia in SCD are secondary to UTI [44]. Moreover, UTIs are more frequent during pregnancy in women with SCD or sickle cell trait [6–8], with increasing prevalence of bacteriuria and UTI according to HbS levels [43, 45, 46].

However, clinical studies have shown that SCD patients present disturbances in the urinary bladder, such as enuresis [14, 47]. The lower urinary tract consists of the urinary bladder and urethra. The urethra contributes to urinary continence by relaxing during the voiding phase and contracting during urine storage phase. Urethral smooth muscle is mainly innervated by sympathetic fibers, occasioning in the release of noradrenaline that acts on postjunctional α1-adrenoceptors and leading to urethral contractions. This excitatory pathway is responsible for the DSM contraction that maintains continence [25]. We herein show that SS mice exhibit a contractile response reduction of the urethra, leading to impaired urinary continence, which may contribute to the enuresis symptoms observed in the SCD patients.

One can expect that the urological complaints caused by SCD-associated UBD are underreported and may be confused with symptoms of more prevalent urological disorders whose incidence increases with age, such as urinary stress incontinence and benign prostatic hyperplasia, particularly with this patient population’s increasing survival [4]. This might hinder the ability of clinical observations to detect UBD specifically associated with SCD. Moreover, UBD is a common but poorly understood lower urinary tract dysfunction that affects both genders [48], and this animal model may contribute to further research on all aspects of UBD.

## Conclusions

In summary, this study is the first to perform a detailed *ex vivo* evaluation of the urethra- and detrusor-muscle functional response and urinary function in SS mice, showing evidence to indicate the presence of a SCD-associated UBD caused by an atonic detrusor smooth muscle, resulting in underactive bladder and the impairment of emptying efficacy. Finally, this is the first experimental evidence of SCD-associated UBD and data suggest that both morphological- and urinary continence-alterations may contribute to UTIs and the enuresis reported in SCD.
